# Candidate Genes and Favorable Haplotypes Associated with Iron Toxicity Tolerance in Rice

**DOI:** 10.3390/ijms25136970

**Published:** 2024-06-26

**Authors:** Siyu Miao, Jingbing Lu, Guogen Zhang, Jing Jiang, Pingping Li, Yukang Qian, Wensheng Wang, Jianlong Xu, Fan Zhang, Xiuqin Zhao

**Affiliations:** 1 State Key Laboratory of Crop Gene Resources and Breeding, Institute of Crop Sciences, Chinese Academy of Agricultural Sciences (CAAS), Beijing 100081, China; miaosiyu1998@163.com (S.M.); 13655060001@163.com (J.L.); jiangjing7199@163.com (J.J.); lipingpingmn@163.com (P.L.); 15997852438@163.com (Y.Q.); wangwensheng02@caas.cn (W.W.); xujianlong@caas.cn (J.X.); 2 College of Agronomy, Anhui Agricultural University, Hefei 230036, China; fruitroot@163.com

**Keywords:** Fe toxicity tolerance, GWAS, physiological characteristics, candidate genes, rice

## Abstract

Iron (Fe) toxicity is a major issue adversely affecting rice production worldwide. Unfortunately, the physiological and genetic mechanisms underlying Fe toxicity tolerance in rice remain relatively unknown. In this study, we conducted a genome–wide association study using a diverse panel consisting of 551 rice accessions to identify genetic mechanisms and candidate genes associated with Fe toxicity tolerance. Of the 29 quantitative trait loci (QTL) for Fe toxicity tolerance detected on chromosomes 1, 2, 5, and 12, five (*qSH_Fe5*, *qSFW_Fe2.3*, *qRRL5.1*, *qRSFW1.1*, and *qRSFW12*) were selected to identify candidate genes according to haplotype and bioinformatics analyses. The following five genes were revealed as promising candidates: *LOC_Os05g40160*, *LOC_Os05g40180*, *LOC_Os12g36890*, *LOC_Os12g36900*, and *LOC_Os12g36940*. The physiological characteristics of rice accessions with contrasting Fe toxicity tolerance reflected the importance of reactive oxygen species–scavenging antioxidant enzymes and Fe homeostasis for mitigating the negative effects of Fe toxicity on rice. Our findings have clarified the genetic and physiological mechanisms underlying Fe toxicity tolerance in rice. Furthermore, we identified valuable genetic resources for future functional analyses and the development of Fe toxicity–tolerant rice varieties via marker–assisted selection.

## 1. Introduction

Iron (Fe), which is an essential element in plants, affects diverse physiological processes, including chlorophyll biosynthesis, photosynthesis, respiration, electron transport, and redox reactions [1,2]. Iron has two common valence states: ferric Fe (Fe^3+^) and ferrous Fe (Fe^2+^). The redox cycling between the two valence states is critical for Fe uptake and translocation in plants, but it also contributes to Fe toxicity because of the associated generation of reactive oxygen species (ROS) [3]. To acquire enough Fe while avoiding its toxic effects, plants have evolved two distinct strategies [4]. Strategy I, which is used by all plants except graminaceous plants, involves the release of protons into the rhizosphere to lower the soil pH as well as the induction of Fe^3+^–chelate reductase gene expression to reduce Fe^3+^ to the more soluble Fe^2+^ form, which is then transported into cells through an iron–regulated transporter (IRT) [5]. Graminaceous plants apply Strategy II, which involves the synthesis and release of mugineic acid family phytosiderophores (MAs) into the soil. Fe^3+^ in the rhizosphere can form chelates with MAs and be absorbed by cells via YS1 (yellow stripe 1) and YSL (yellow stripe1–like) transporters. Although rice (*Oryza sativa* L.) is considered to be a Strategy II plant, it has adapted to grow under submerged conditions, with a relatively high Fe^2+^ content in the rhizosphere. Therefore, rice has evolved a mechanism for absorbing Fe^2+^. Specifically, Fe^2+^ in soil is transported into rice cells mainly through *OsIRT1* and *OsIRT2* [6]. However, the absorption of excessive amounts of Fe^2+^ from acidic soil increases leaf cell Fe^2+^ concentrations to toxic levels [7]. Rice can grow optimally in soil with an Fe concentration less than 300 mg L^−1^. However, at concentrations exceeding 300 mg L^−1^, rice plants exhibit symptoms of Fe toxicity. Under severely toxic conditions, the Fe concentration may be as high as 1000 mg L^−1^ [8]. Fe toxicity can decrease rice yields by 10–90% in major rice–producing regions worldwide [9,10], including Asia and parts of West Africa [11,12,13,14,15].

Hallmark symptoms of Fe toxicity in rice include the “bronzing” of leaves and stunted root growth [16]. The toxicity of Fe is primarily due to the generation of ROS and hydroxyl radicals (·OH) via the Fenton reaction, resulting in cellular damage and disrupted physiological processes [17]. Furthermore, Fe excess can hinder the uptake of other cations by roots [18]. To cope with Fe toxicity, rice plants have evolved at least three tolerance mechanisms: (1) exclusion of Fe^2+^ at the root level and avoidance of Fe^2+^ damage to the shoot tissue [19,20]; (2) storage of excessive Fe in the apoplast or vacuole as well as adsorption of Fe by ferritin in plastids [21,22]; and (3) enzymatic detoxification of ROS [23].

Rice’s tolerance to Fe toxicity is a complex trait controlled by multiple genes. Numerous studies have identified many main–effect quantitative trait loci (QTLs) [24,25,26,27,28,29,30,31,32,33,34,35,36,37,38,39] and epistatic QTLs [40,41,42] for rice’s tolerance to Fe toxicity under varying conditions. For example, Liu et al. [38] identified nine QTLs associated with rice’s resistance to Fe toxicity on the basis of bidirectional imported line populations derived from MH63 and 02428. Meng et al. [39] used a rice MAGIC population to analyze nine traits associated with Fe toxicity tolerance, resulting in the detection of 30 QTLs related to Fe toxicity tolerance. In addition, some genes contributing to the rice response to Fe toxicity have been identified. Both *OsFER1* and *OsFER2* may be involved in the resistance of rice to Fe–mediated oxidative stress [43], whereas *OsVIT1* and *OsVIT2* encode proteins that transport excess zinc and Fe ions to vacuoles and maintain intracellular Fe homeostasis [21]. Additionally, *OsFRO1* is important for maintaining Fe homeostasis between the cytoplasm and vacuole in rice [44]. Genome–wide association study (GWAS)–based research has recently been conducted to identify QTLs/genes associated with Fe toxicity tolerance in different rice populations [45,46,47,48,49,50,51]. Matthus et al. [45] conducted a GWAS involving 329 rice accessions and concluded that the maintenance of foliar redox homeostasis under Fe stress conditions is important for Fe toxicity tolerance. Li et al. [46] identified S–nitrosoglutathione–reductase gene (*GSNOR*) variants associated with a major QTL for Arabidopsis root tolerance to Fe toxicity on the basis of a GWAS and allelic complementation. Zhang et al. [47] used 222 *indica* rice germplasm to conduct a GWAS and identified 14 QTLs for rice tolerance to Fe toxicity. Diop et al. [48] identified promising GWAS signals and putative candidate genes involved in the response to high–Fe stress. Although some QTLs for Fe toxicity tolerance have been identified in rice, the genetic basis of Fe toxicity tolerance in the *Geng*/*Japonica* (*GJ*) subpopulation and the differences between *Xian*/*Indica* (*XI*) and *GJ* subpopulations remain unknown. In this study, we performed a GWAS using 551 accessions from the 3000 Rice Genomes Project (3K–RG) [52] to clarify Fe toxicity tolerance in rice. We also compared the physiological responses of representative Fe toxicity–tolerant and Fe toxicity–sensitive accessions to Fe toxicity. Our findings elucidate the complex mechanisms underlying Fe toxicity tolerance and may be relevant to future research on the functional characterization of genes and the genetic improvement of rice.

## 2. Results

### 2.1. Phenotypic Changes Related to Fe Toxicity Tolerance

**Figure 1:**
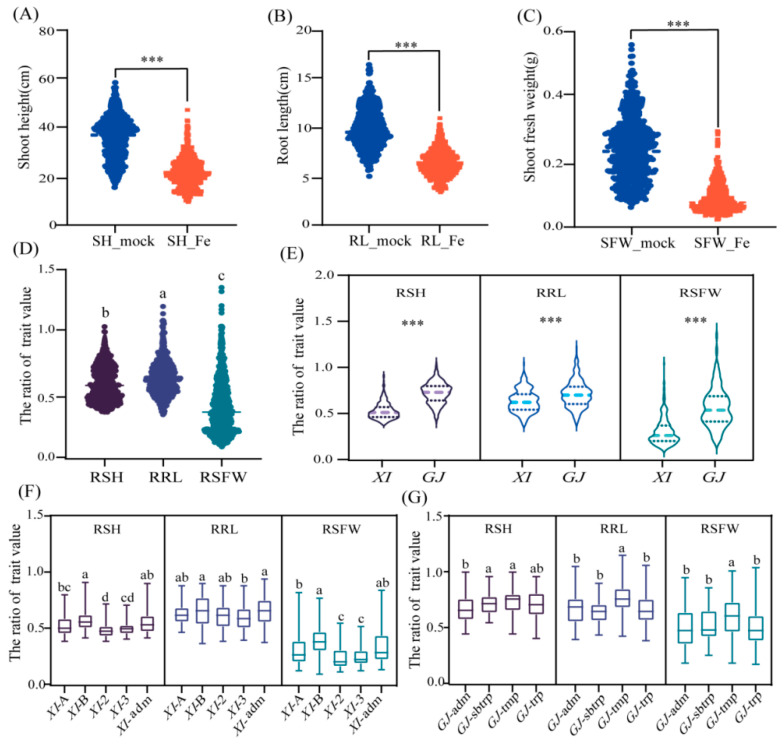
Phenotypic changes caused by Fe toxicity. Distribution of the (**A**) shoot height (SH), (**B**) root length (RL), and (**C**) shoot fresh weight (SFW) following the control and Fe toxicity treatments. (**D**) Distribution of the ratios of trait values in the whole population. (**E**) Distribution of the ratios of trait values in the *XI* and *GJ* subpopulations. (**F**) Distribution of the ratios of trait values for the *XI*-1A, *XI*-1B, *XI*-2, *XI*-3, and *XI*-adm accessions. (**G**) Distribution of the ratios of trait values for the *GJ*-adm, *GJ*-subtropical (*GJ*-sbtrp), *GJ*-temperate (*GJ*-tmp), and *GJ*-tropical (*GJ*-trp) accessions. For the Fe toxicity (‘trait name_Fe’) and control (‘trait name_mock’) treatments, the ratios of trait values (R + ‘trait name’) are provided. *** indicates a significant difference (*p* < 0.001) determined by an analysis of variance (ANOVA). Different letters above each histogram indicate significant differences among traits (*p* < 0.05) determined by Duncan’s multiple range test.

A total of 551 rice accessions from 3K–RG (279 *XI* accessions and 272 *GJ* accessions) were evaluated for phenotypic changes due to Fe toxicity (Table S1). A 20-day treatment with 300 mg L^−1^ FeSO_4_·7H_2_O decreased the shoot height (SH), root length (RL), and shoot fresh weight (SFW) by 38.8%, 34.0%, and 62.5%, respectively (Figure 1A–C). According to the ratios of the trait values for the Fe toxicity and control treatments, the severity of the effect of Fe toxicity varied widely in the whole population. Notably, among these three traits, SFW was affected the most by Fe toxicity (Figure 1D). The ratios of shoot height (RSH), root length (RRL), and shoot fresh weight (RSFW) were significantly higher for the *GJ* accessions than for the *XI* accessions (Figure 1E). Of the *XI* subgroups, *XI*-B and *XI*-adm were the most tolerant to Fe toxicity (Figure 1E). Among the four *GJ* subgroups, *GJ*-tmp included the most accessions which were tolerant to Fe toxicity (Figure 1G). Significant positive correlations were detected between several phenotypic traits, including between SH_Fe and SFW_Fe (correlation coefficient of 0.82) and between RSH and RSFW (correlation coefficient of 0.85), in the whole population. Interestingly, the correlation coefficients among the six phenotypic traits were higher for the *GJ* accessions than for the *XI* accessions (Figure S1).

### 2.2. Morphological and Physiological Differences between Accessions with Contrasting Fe Toxicity Tolerance

**Figure 2:**
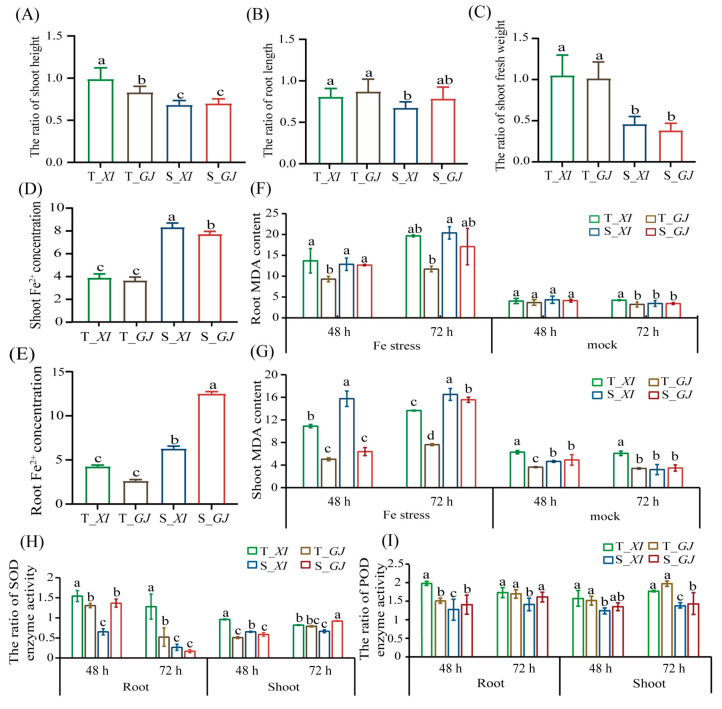
Morphological and physiological differences between accessions with contrasting Fe toxicity tolerance. (**A**–**C**) Ratios of trait values for the four tolerant and sensitive accessions. RSH, ratio of shoot height; RRL, ratio of root length; RSFW, ratio of shoot fresh weight. Ratios of the Fe^2+^ concentrations in the shoots (**D**) and roots (**E**). MDA contents in the roots (**F**) and shoots (**G**) after 48 h and 72 h Fe toxicity and control treatments. Ratios of SOD (**H**) and POD (**I**) activities in the roots and shoots. The green, brown, blue and red column represents T_*XI*, T_*GJ*, S_*XI*, and S_*GJ*, respectively. Data are presented as the mean and standard deviation. Different letters above the histogram indicate significant differences (*p* < 0.05) as determined by Duncan’s multiple range test.

To examine the response of rice accessions to Fe toxicity, we selected two tolerant accessions (*XI* accession Tun Sart (T_*XI*) and *GJ* accession SHINCHIKU–IKU 97 (T_*GJ*)) and two sensitive accessions (*XI* accession NCS766 (S_*XI*) and *GJ* accession KETAN LALER (S_*GJ*)) on the basis of RSH, RRL, and RSFW values (Table S1, Figure S2). The SH, RL, and SFW values decreased in T_*XI* (by 41.6%, 34.1%, and 62.8%, respectively), T_*GJ* (by 35.1%, 4.8%, and 50.8%, respectively), S_*XI* (by 58.7%, 54.3%, and 91.7%, respectively), and S_*GJ* (by 32.8%, 39.8%, and 46.2%, respectively) (Figure 2A–C). After exposure to Fe toxicity, the Fe^2+^ concentrations in the shoots and roots were significantly higher in the sensitive accessions (S_*XI* and S_*GJ*) than in the tolerant accessions (T_*XI* and T_*GJ*); the differences were greater for the roots than for the shoots (Figure 2D,E). To determine the changes to the cell membrane and redox homeostasis under Fe toxicity stress conditions, we compared the physiological traits of the tolerant and sensitive accessions after 48 h and 72 h treatments. The comparisons with the tolerant accessions revealed that the malondialdehyde (MDA) contents in the roots and shoots were significantly higher in the sensitive accessions at the 72 h time point of the Fe toxicity treatment (Figure 2F,G). Conversely, the superoxide dismutase (SOD) activity in the roots was significantly lower in the sensitive accessions than in the tolerant accessions at 72 h, possibly reflecting the inhibitory effects of Fe on SOD (Figure 2H). Peroxidase (POD) activity was significantly higher in the shoots of the tolerant accessions than in the shoots of the sensitive accessions (Figure 2I). Our results suggest that ROS–scavenging antioxidant enzymes and Fe homeostasis are important for Fe toxicity tolerance in rice.

### 2.3. GWAS for Traits Related to Fe Toxicity Tolerance

**Figure 3:**
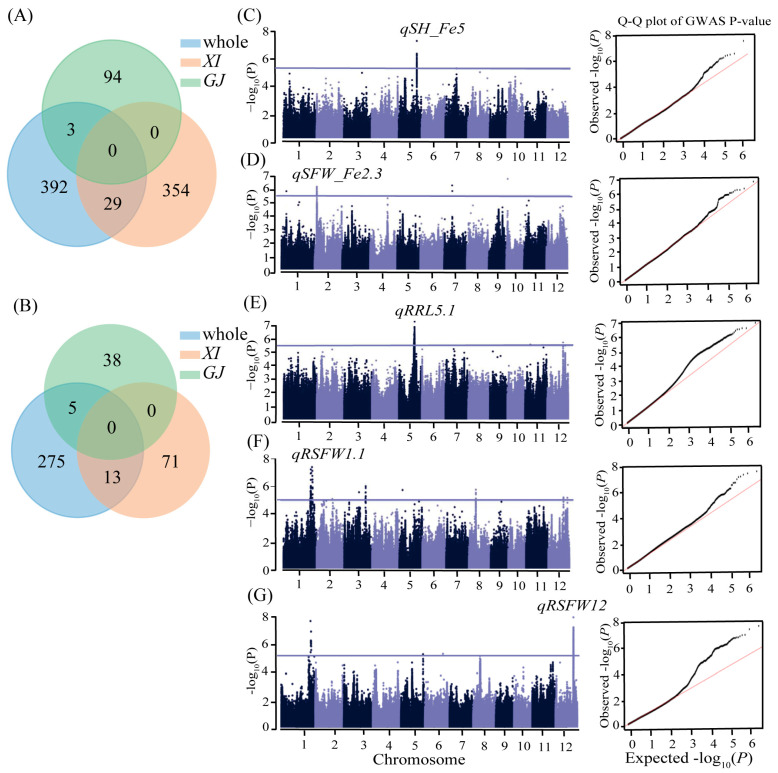
Results of GWAS for Fe toxicity tolerance. The Venn diagram shows the numbers of common SNPs (**A**) and genes (**B**) significantly associated with Fe toxicity tolerance in the different populations. Manhattan and QQ plots of GWAS results for SH_Fe in the *GJ* subpopulation (**C**), for SFW_Fe in *XI* subpopulation (**D**), for RRL in whole population (**E**), for RSFW in whole population (**F**), and in the *XI* subpopulation (**G**).

On the basis of a principal component analysis and kinship analysis, the 551 rice accessions were clustered into distinct subpopulations (*XI* and *GJ*) (Figure S3). We conducted a GWAS involving six traits related to Fe toxicity tolerance (SH_Fe, RL_Fe, SFW_Fe, RSH, RRL, and RSFW) for the whole population as well as the *XI* and *GJ* subpopulations (Figures S4–S6). In total, 424, 383, and 97 single–nucleotide polymorphisms (SNPs) significantly associated with Fe toxicity tolerance were identified in the whole population, *XI* subpopulation, and *GJ* subpopulation, respectively, among which 29 SNPs were shared between the whole population and the *XI* subpopulation, whereas three SNPs were shared between the whole population and the *GJ* subpopulation (Figure 3A). The subsequent analysis of the genes associated with the significant SNPs in the three populations revealed 13 overlapping genes between the whole population and the *XI* subpopulation, 5 overlapping genes between the whole population and the *GJ* subpopulation, and no overlapping genes between the *XI* and *GJ* subpopulations (Figure 3B). Finally, 29 QTLs associated with Fe toxicity tolerance were identified in the three GWAS panels, of which 19 were detected under Fe toxicity stress conditions, whereas the other 10 were detected on the basis of the ratios of trait values (Table S2). We selected five important QTLs (*qSH_Fe5*, *qSFW_Fe2.3*, *qRRL5.1*, *qRSFW1.1*, and *qRSFW12*), each with more than 30 significant SNPs, for further analyses of candidate genes (Table S3).

### 2.4. Candidate Gene Analysis

**Figure 4:**
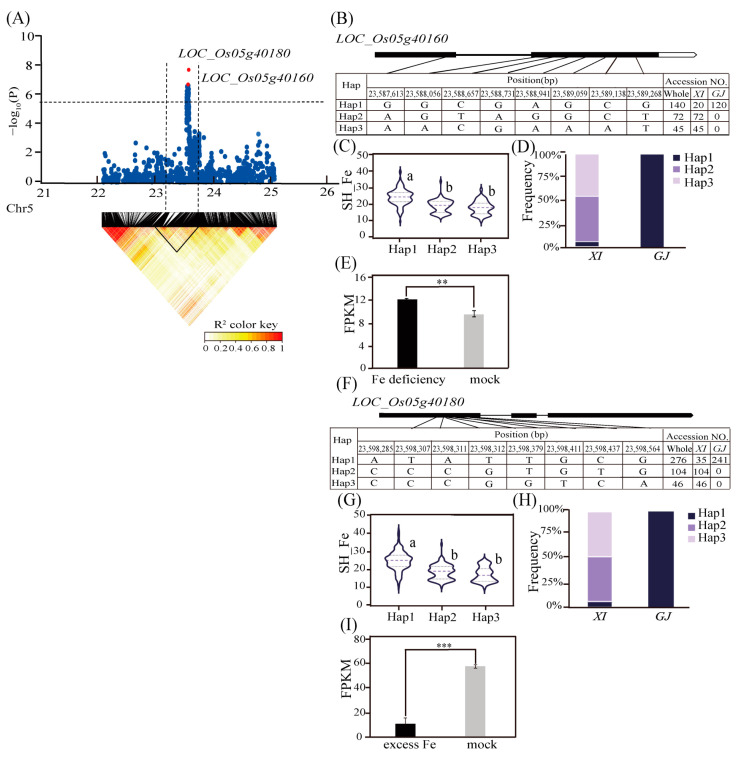
Candidate gene analysis for *qSH_Fe5*. (**A**) Local Manhattan plot (upper) and LD heatmap (lower) surrounding the lead SNP of *qSH_Fe5*. (**B**) Haplotype analysis of *LOC_Os05g40160*. (**C**) Differences in SH_Fe among the haplotypes of *LOC_Os05g40160*. (**D**) Frequency of three haplotypes of *LOC_Os05g40160* in the *XI* and *GJ* subpopulations. (**E**) *LOC_Os05g40160* expression levels under Fe–deficient and control conditions. Data were obtained from the Plant Public RNA–Seq Database. The rice material was *O. sativa* cv *Nipponbare*. (**F**) Haplotype analysis of *LOC_Os05g40180*. (**G**) Differences in SH_Fe among the haplotypes of *LOC_Os05g40180*. (**H**) Frequency of three haplotypes of *LOC_Os05g40180* in the *XI* and *GJ* subpopulations. (**I**) *LOC_Os05g40180* expression levels under Fe toxicity and control conditions. Data were obtained from the Plant Public RNA–seq Database. The *indica* rice varieties were Hacha and Lachit. ** indicates a highly significant difference (*p* < 0.01), *** indicates a significant difference (*p* < 0.001). Different letters above each boxplot indicate significant differences (*p* < 0.05) among haplotypes as determined by Duncan’s multiple range test.

Of the 12 candidate genes identified for Fe toxicity tolerance in the five important QTLs, five were associated with abiotic stress responses, and their expression could be induced by Fe excess or deficiency. For *qSH_Fe5*, the predicted linkage disequilibrium (LD) block region on chromosome 5 (23.45–23.75 Mb) included *LOC_Os05g40180* (Figure 4A), which was associated with a lead SNP (rs5_23597337; *p* = 1.92 × 10^−8^) for SH_Fe in the *GJ* subpopulation. This gene encodes a serine/threonine protein kinase, which belongs to a large family of enzymes involved in environmental stress signal transduction [53,54]. Three major haplotypes (*n* ≥ 30 accessions) of *LOC_Os05g40180* were detected on the basis of eight SNPs in the coding sequence (CDS) region (Figure 4F). The SH_Fe values differed significantly across the three haplotypes (i.e., significantly higher for Hap1 than for Hap2 and Hap3) (Figure 4G). Hap1 was the predominant haplotype in the *GJ* subpopulation, whereas Hap2 and Hap3 were mainly enriched in the *XI* subpopulation (Figure 4H). In addition, according to the Plant Public RNA–seq Database (http://ipf.sustech.edu.cn/pub/plantrna/, accessed on 23 February 2024), *LOC_Os01g40180* expression was significantly down–regulated in response to a 2-day Fe toxicity treatment.

*LOC_Os01g40160*, which was identified as another candidate gene for *qSH_Fe5*, encodes a protein with a nucleotide–binding adapter shared by APAF–1, R proteins, and the CED–4 (NB–ARC) domain. We performed a haplotype analysis using eight SNPs in the CDS region and identified three major haplotypes (Figure 4B). Among the major haplotypes, Hap1, which had a significantly higher SH_Fe value and was considered to be the favorable haplotype (Figure 4C), was significantly enriched in *GJ* accessions (Figure 4D). According to the Plant Public RNA–seq Database (http://ipf.sustech.edu.cn/pub/plantrna/, accessed on 23 February 2024), *LOC_Os05g40160* expression was significantly up–regulated under Fe–deficient conditions (Figure 4E).

**Figure 5:**
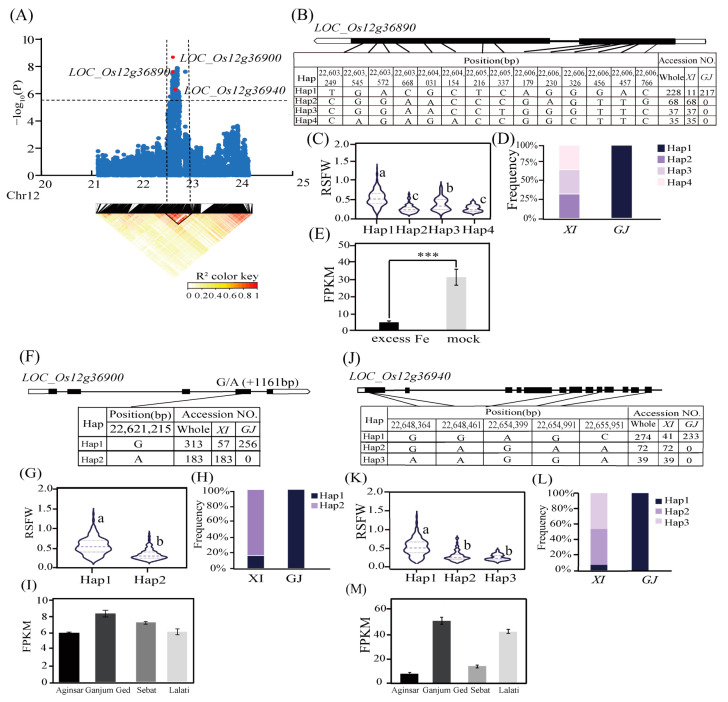
Candidate gene analysis for *qRSFW12*. (**A**) Local Manhattan plot (upper) and LD heatmap (lower) surrounding the lead SNP for *qRSFW12*. (**B**) Haplotype analysis of *LOC_Os12g36890*. (**C**) Differences in RSFW among the haplotypes of *LOC_Os12g36890*. (**D**) Frequency of three *LOC_Os12g36890* haplotypes in the *XI* and *GJ* subpopulations. (**E**) *LOC_Os12g36890* expression levels under Fe toxicity and control conditions. Data were obtained from the Plant Public RNA–Seq Database. The indica *rice* varieties were Hacha and Lachit. (**F**) Haplotype analysis of *LOC_Os12g36900*. (**G**) Differences in RSFW among the haplotypes of *LOC_Os12g36900*. (**H**) Frequency of three *LOC_Os12g36900* haplotypes in the *XI* and *GJ* subpopulations. (**I**) *LOC_Os12g36900* expression levels in the tolerant accessions (Ganjum Ged and Lalati) and sensitive accessions (Aginsar and Sebat). Data were obtained from the Plant Expression Portal Database (SRP188072). (**J**) Haplotype analysis of *LOC_Os12g36940*. (**K**) Differences in RSFW among the haplotypes of *LOC_Os12g36940*. (**L**) Frequency of three *LOC_Os12g36940* haplotypes in the *XI* and *GJ* subpopulations. (**M**) *LOC_Os12g36940* expression levels in the tolerant accessions (Ganjum Ged and Lalati) and sensitive accessions (Aginsar and Sebat). Data were obtained from the Plant Expression Portal Database (SRP188072). *** indicates an extremely significant difference (*p* < 0.001). Different letters above each boxplot indicate significant differences (*p* < 0.05) among haplotypes as determined by Duncan’s multiple range test.

For *qRSFW12*, the LD block region on chromosome 12 (22.48–22.75 Mb) included three candidate genes for Fe toxicity tolerance (*LOC_Os12g36890*, *LOC_Os12g36900*, and *LOC_Os12g36940*) (Figure 5A). *LOC_Os12g36900* harbored the most significant SNP (rs12_22616855; *p* = 2.09 × 10^−9^) for RSFW in the *XI* subpopulation. A missense variant at 22,621,215 bp was detected between Hap1 and Hap2 (Figure 5F); RSFW was significantly higher for haplotype ‘G’ than for haplotype ‘A’ (Figure 5G). Hap1 was mainly enriched in the *GJ* subpopulation (Figure 5H). According to the Plant Expression Portal Database (https://biotec.njau.edu.cn/plantExp/index.php, accessed on 23 February 2024), the *LOC_Os12g36900* expression level was significantly higher in the tolerant accessions (Ganjum Ged and Lalati) than in the sensitive accessions (Aginsar and Sebat) following exposure to Fe toxicity stress (Figure 5I). The SNP (rs12_22611603) located in the candidate gene *LOC_Os12g36890* was significantly associated with RSFW in the *XI* subpopulation. *LOC_Os12g36890*, which is also known as *OsCSLD4* or *NRL1*, encodes a cell wall cellulose synthase–like D4 protein important for the response to salt stress. More specifically, it mediates abscisic acid biosynthesis to regulate osmotic stress tolerance in rice [55]. Using 14 SNPs in the CDS region of *LOC_Os12g36890*, we identified four major haplotypes, each of which was detected in at least 30 accessions (Figure 5B). Compared with the other haplotypes, Hap1 had a significantly higher RSFW (Figure 5C,D). Moreover, it was mainly present in *GJ* accessions. The expression of *LOC_Os12g36890* was significantly down–regulated after 2 days of the Fe toxicity treatment according to the Plant Public RNA–Seq Database (http://ipf.sustech.edu.cn/pub/plantrna/, accessed on 23 February 2024) (Figure 5E). A haplotype analysis of *LOC_Os12g36940*, which encodes a calmodulin–binding protein, showed that Hap1 had a significantly higher RSFW than Hap2 and Hap3 and was mainly enriched in the *GJ* subpopulation (Figure 5J–L). According to the transcriptome data in the Plant Expression Portal Database (https://biotec.njau.edu.cn/plantExp/index.php, accessed on 23 February 2024), the *LOC_Os12g36940* expression level was 3-fold higher in the tolerant accessions (Ganjum Ged and Lalati) than in the sensitive accessions (Aginsar and Sebat) under Fe toxicity stress conditions (Figure 5M). Considered together, these results suggest that *LOC_Os01g40160*, *LOC_Os01g40180*, *LOC_Os12g36890*, *LOC_Os12g36900*, and *LOC_Os12g36940* may be important candidate genes modulating Fe toxicity tolerance in rice.

## 3. Discussion

In plants, Fe toxicity disrupts diverse biological processes, including photosynthesis, respiration, and nitrogen assimilation, ultimately leading to cell damage [56]. Rice’s tolerance to Fe toxicity is a complex trait controlled by multiple genes. Analyses of genetic populations derived from crosses between sensitive and tolerant rice varieties have resulted in the identification of numerous QTLs related to Fe toxicity tolerance (Table S4). In the current study, 30% of the identified loci for Fe toxicity tolerance were co-located with previously reported QTLs [35,39,47], including *qSH_Fe7*, *qSFW_Fe2.1*, *qSFW_Fe3.1*, *qSFW_Fe12*, *qRSFW3*, *qRSH1.2*, *qRRL7*, and *qRSFW1.2* (Table S2). A comprehensive analysis of significant SNPs, haplotypes, gene expression, and functional annotations revealed five promising candidate genes (*LOC_Os05g40160*, *LOC_Os05g40180*, *LOC_Os12g36890*, *LOC_Os12g36900*, and *LOC_Os12g36940*) within five significant QTLs (Figure 4 and Figure 5). Notably, *LOC_Os05g40160*, the most likely candidate gene for *qSH_Fe5*, encodes a protein that includes an NB–ARC domain, which is critical for regulating signaling pathways involved in effector recognition and signal transduction essential for plant growth and development [57,58,59,60,61]. *LOC_Os05g40180* (*OsSTN8*), which was identified as another important candidate gene of *qSH_Fe5*, encodes a serine/threonine protein kinase contributing to photosystem II (PSII) core protein phosphorylation. In the *osstn8* mutant, ROS accumulates, and PSII reaction center core proteins in thylakoid membranes are preferentially oxidized under high–light conditions [62]. In the present study, we identified the following three promising candidate genes for *qRSFW12*: *LOC_Os12g36890* (*OsCSLD4*), *LOC_Os12g36900* (*OsDi19*-*7*) and *LOC_Os12g36940*. Among the encoded proteins, *OsCSLD4* regulates cell wall polysaccharide synthesis and is important for salt tolerance because it mediates abscisic acid biosynthesis [55]. *OsDi19*-*7* belongs to the drought–induced 19 (Di19) protein family. Of the seven Di19 genes in the rice genome, *OsDi19*-*4* is involved in drought tolerance [63]. *LOC_Os12g36940* encodes a calmodulin–binding protein, suggesting it may affect calcium–mediated signaling in response to excessive Fe levels [64]. In plant cells, Ca^2+^ is a second messenger that participates in various biotic and abiotic stress responses [65,66,67]. Sun et al. [68] determined that Ca^2+^ and the mitogen–activated protein kinase cascade regulate the signaling associated with the response of apple root to Fe deficiency.

There were distinct differences in the responses of the *XI* and *GJ* subpopulations to Fe toxicity. Moreover, *GJ* accessions, especially *GJ*–tmp, were more tolerant to Fe toxicity than *XI* accessions (Figure 1E–G). This finding is in accordance with the results of a similar study by Matthus et al. [45], which showed that aromatic and temperate *GJ* accessions had lower shoot Fe^2+^ concentrations and leaf bronzing scores than *XI* accessions under Fe toxicity stress conditions. In this study, there were no common association signals between the *XI* and *GJ* subpopulations (Figure 3B), suggesting that the genes underlying Fe toxicity tolerance and/or the distribution of functional alleles may differ significantly between the two rice subspecies. Furthermore, favorable haplotypes of the candidate genes for Fe toxicity tolerance were mainly carried by *GJ* accessions, including *LOC_Os05g40160*^Hap1^, *LOC_Os05g40180*^Hap1^, *LOC_Os12g36890*^Hap1^, *LOC_Os12g36900*^Hap1^, and *LOC_Os12g36940*^Hap1^. Considered together, these results imply that the molecular mechanism mediating rice’s Fe toxicity tolerance varies among subspecies.

Oxidative stress caused by Fe toxicity can induce the formation of free radicals, with detrimental effects on crucial biomolecules (e.g., proteins and lipids) [46,69]. An increase in the MDA content in plant tissues is an indicator of lipid peroxidation within cells. Under Fe toxicity stress conditions, MDA production increased significantly in rice leaves, ultimately leading to cell death [70]. Awasthi et al. [71] reported that exposure to Fe toxicity stress increases lipid peroxidation and plasma membrane damage in roots. A negative correlation between the leaf chlorophyll and MDA contents has also been reported [72]. Consistent with these previous studies, we detected a significant increase in the MDA content of samples (especially the sensitive accessions) that underwent the Fe toxicity treatments (Figure 2F,G). Regon et al. [73] proposed a role for ROS detoxification in the tolerance to severe Fe toxicity. Exposure to excessive Fe levels enhances the activities of antioxidant enzymes, such as SOD, catalase (CAT), ascorbate peroxidase, glutathione reductase (GR), and POD, in rice [74,75]. For example, Fe toxicity can significantly increase the activities of CAT, POD, GR, and SOD in the shoots of the rice variety Pokkali, thereby enhancing antioxidative defenses [76]. Earlier research has also demonstrated that POD activity is induced by Fe^2+^ [77,78]. In the current study, POD activity in shoots was significantly higher in the tolerant accessions than in the sensitive accessions (Figure 2I). Thus, the genotype–specific response to Fe toxicity may be partially attributed to differences in antioxidant enzyme activities. Additionally, compared with the shoots, the roots had higher Fe^2+^ concentrations (Figure 2D,E), implying that the root system is the primary rice tissue affected by Fe toxicity. The ability of Fe toxicity–tolerant accessions to maintain Fe ion homeostasis may also contribute to their tolerance. In future studies, the mechanisms regulating ROS–scavenging antioxidant enzymes and Fe ion homeostasis under Fe toxicity stress conditions will need to be comprehensively investigated.

The use of Fe toxicity–tolerant varieties is an effective strategy for mitigating the harmful effects of Fe toxicity on rice yield and quality. Several studies have assessed rice’s tolerance to Fe toxicity under various conditions, resulting in the identification of valuable genetic resources for breeding programs attempting to enhance Fe toxicity tolerance. For example, Streck et al. [79] identified 15 tolerant genotypes by evaluating the responses of rice varieties from the Embrapa breeding program to Fe stress. Similarly, Ahmed et al. [8] identified an Fe toxicity–tolerant rice genotype (RD85) by analyzing the stress tolerance indices of different rice genotypes. According to the results of the present study, T_*XI* and T_*GJ* are two germplasm resources which are highly tolerant to Fe toxicity, making them potentially useful donor parents for the breeding of novel rice varieties. We analyzed the haplotypes of five candidate genes in these two accessions (Table S5). Interestingly, T_*XI* carries Hap2 of *LOC_Os12g36900*, whereas T_*GJ* carries the favorable haplotype (Hap1) of *LOC_Os05g40180*. The specific haplotypes of three candidate genes are presented in Figure 4 and Figure 5. Furthermore, five QTLs (*qSH_Fe5*, *qSFW_Fe2.3*, *qRRL5.1*, *qRSFW1.1,* and *qRSFW12*) may be used to improve rice’s tolerance to Fe toxicity via marker–assisted selection or QTL pyramiding. Specifically, to increase the tolerance of *XI* varieties, the *GJ*-specific favorable alleles/haplotypes of candidate genes should be introgressed into the *XI* genetic background through inter–subspecific crosses. Future research should focus on validating and functionally characterizing important candidate genes (*LOC_Os05g40160*, *LOC_Os05g40180*, *LOC_Os12g36890*, *LOC_Os12g36900*, and *LOC_Os12g36940*) using transgenic materials. This will enhance our understanding of the mechanisms underlying Fe toxicity tolerance in rice, with implications for designing more effective breeding strategies.

## 4. Materials and Methods

### 4.1. Plant Materials

The 551 rice accessions from 3K–RG [52] used for the GWAS included 279 *XI* accessions and 272 *GJ* accessions (Table S1). Two representative Fe toxicity–tolerant accessions (T_*XI* and T_*GJ*) and two representative Fe toxicity–sensitive accessions (S_*XI* and S_*GJ*) were selected for an analysis of physiological characteristics.

### 4.2. Growth Conditions and Phenotyping Examination

The phenotypes of the 551 selected rice accessions were analyzed in a greenhouse at the Institute of Crop Sciences of the Chinese Academy of Agricultural Sciences (Beijing, China) from September to October 2021. Before sowing, 40–50 dormancy–breaking seeds of each accession were surface–sterilized in a 5% sodium hypochlorite solution (NaOCl) for 30 min and then rinsed thoroughly with distilled water. The rinsed seeds were then immersed in distilled water for 72 h at 30 °C. Eight germinated seeds per accession were sown in a 96–well PCR plate with wells that had perforated bottoms. The plates with seeds were maintained in tap water for 5 days and then transferred to Yoshida nutrient solution [80]. The nutrient solution contained 40 mg L^−1^ (NH_4_)_2_SO_4_, 10 mg L^−1^ NaH_2_PO_4_·2H_2_O, 40 mg L^−1^ K_2_SO_4_, 40 mg L^−1^ CaCl_2_·2H_2_O, 40 mg L^−1^ MgSO_4_·7H_2_O, 0.5 mg L^−1^ MnCl_3_·4H_2_O, 0.05 mg L^−1^ (NH_4_)_6_Mo_7_O_24_·4H_2_O, 0.01 mg L^−1^ ZnSO_4_·7H_2_O, 0.2 mg L^−1^ H_3_BO_3_, 0.01 mg L^−1^ CuSO_4_·5H_2_O, 2 mg L^−1^FeCl_3_·6H_2_O, and 11.9 g L^−1^ C_6_H_8_O_7_·H_2_O. Nutrient solution required chemical reagents are from Xilong Scientific Co., Ltd, Shenzhen City, China. The plates were incubated in a greenhouse set at approximately 28 °C (day)/25 °C (night) with a relative humidity of approximately 60%. The nutrient solution (pH maintained at 5.5 ± 0.5) was replaced every 5 days. After a 10-day growth period, the seedlings underwent the Fe toxicity and control treatments for 20 days. For the Fe toxicity treatment, the Yoshida nutrient solution was supplemented with 300 mg L^−1^ (5.36 mM) FeSO_4_·7H_2_O, which was higher than the FeSO_4_·7H_2_O concentration (2.0 mg L^−1^) of the Yoshida nutrient solution used for the control treatment. Both treatments were completed using three replicates. After a 20-day treatment period, SH, RL, and SFW were measured. To assess the relative performance of each trait, the following formula was used: ratio of the trait value = trait value for the Fe toxicity treatment/trait value for the control treatment.

### 4.3. Measurement of Fe Ion Concentration

The Fe concentrations in the shoot and root samples under stress conditions were determined using a wet digestion method (GB/T 14609–2008) [81] and an atomic absorption spectrometer (AAS, Series 2, Thermo Electron Corporation, Waltham, MA, USA). The shoot and root samples of the four rice accessions were dried to a constant weight at 80 °C for 3 days and then cut into smaller pieces. The dried tissues (0.1 g) were digested with 0.1 N (100 mM) glacial acetic acid for 2 h at 90 °C. After cooling, the digested samples were diluted with distilled water. A standard solution ion concentration curve was constructed using a standard solution containing 1000 μg mL^−1^ Fe (NCS company, China Iron and Steel Research Institute, Beijing, China).

### 4.4. Measurement of Malondialdehyde Content and Enzyme Activity

The shoots and roots of the four rice accessions that underwent the Fe toxicity and control treatments for 48 and 72 h were collected and immediately frozen using liquid nitrogen. The MDA, SOD, and POD contents were measured using commercial kits (Grace Biotechnology Co., Ltd, Suzhou City, China). Three biological replicates were analyzed.

### 4.5. Genome–Wide Association Study

The genotypes of the 551 rice accessions were determined using the 3K–RG 4.8 M SNP dataset [82], which was filtered using PLINK 1.9 (missing rate < 20% and minor allele frequency > 5%) [83]. Totals of 2,955,964, 2,248,239, and 1,465,040 SNPs were retained for the GWAS of the whole population, *XI* subpopulation, and *GJ* subpopulation, respectively. The GWAS was conducted using a mixed linear model and the Efficient Mixed–Model Association eXpedited (EMMAX) software (http://csg.sph.umich.edu/kang/emmax/download/index, accessed on 23 February 2024) [84]. The kinship matrix was calculated using an identical–by–state matrix and the pruned SNP subset as a measure of the relatedness between accessions. The eigenvectors of the kinship matrix were calculated using GCTA, and the first three principal components were used as covariates to control the population structure [85]. The effective number of independent markers (N) was calculated using the GEC software (http://java.sun.com/javase/downloads/index.jsp, accessed on 23 February 2024) [86]. Additionally, the following suggestive significance thresholds of association were determined according to the Bonferroni correction method (1/N) for significant SNPs: *p* = 2.32 × 10^−6^ for the whole population, *p* = 2.68 × 10^−6^ for the *XI* subpopulation, and *p* = 5.92 × 10^−6^ for the *GJ* subpopulation. Manhattan and Q-Q plots were drawn using the R package “qqman” for R version 4.1.0. [87]. At least 30 significant SNPs within a 300 kb region were considered as QTLs on the basis of the previously reported LD decay in 3K–RG [52]. A local LD block analysis (150 kb upstream and downstream of the leading SNP) was performed using the R package “LD heatmap”for R version 4.1.0. [88].

### 4.6. Identification of Candidate Genes

Candidate genes for Fe toxicity tolerance satisfied at least two of the following criteria: (1) they harbored significant nonsynonymous or regulatory SNPs; (2) they were functionally related to abiotic stress (e.g., heavy metal) tolerance according to Nipponbare reference genome (IRGSP 1.0) annotations [78], Gene Ontology annotations, or literature search; and (3) they were regulated by Fe stress according to the Plant Public RNA–Seq Database [89] and Plant Expression Portal [90]. To analyze gene haplotypes, we used all SNPs in the coding region of an annotated gene. Synonymous SNPs were ignored (merged into one haplotype) [91]. Duncan’s multiple range post hoc tests were completed using the R package “agricolae” to compare the phenotypic differences among haplotypes (*n* ≥ 30 accessions).

### 4.7. Statistical Analyses

We used a one–way analysis of variance (ANOVA) of the R package “agricolae” to analyze the phenotypic differences between subpopulations. Student’s *t*-tests were applied to compare the phenotypic differences between Fe toxicity stress and control conditions, as well as between the *XI* and *GJ* subpopulations. Pearson’s correlation coefficients for different traits were calculated using the R package “corrplot”. Violin plots and boxplots were drawn using the R package “ggplot2”for R version 4.1.0. A Venn diagram was plotted using jvenn (http://jvenn.toulouse.inra.fr/app/example.html, accessed on 20 January 2024). Physiological differences between Fe toxicity–tolerant and Fe toxicity–sensitive accessions were compared using a one–way ANOVA followed by LSD post hoc tests (R version 4.1.0.).

## 5. Conclusions

Rice is consumed by more than half of the global population, but in some regions, the excessive accumulation of Fe in soil has adversely affected rice cultivation. To address this problem, we are currently analyzing rice varieties that can tolerate excessive Fe levels. However, we do not fully understand the physiological and genetic mechanisms underlying Fe toxicity tolerance in rice, which has delayed the development of new Fe–tolerant rice varieties. In this study, we conducted a GWAS involving a diverse panel comprising 551 rice accessions to reveal the molecular mechanisms and candidate genes associated with Fe toxicity tolerance. A total of 29 Fe toxicity tolerance–related QTLs were detected on chromosomes 1, 2, 5, and 12. These QTLs included *qSH_Fe5*, *qSFW_Fe2.3*, *qRRL5.1*, *qRSFW1.1* and *qRSFW12*, which were selected to screen for candidate genes via haplotype and bioinformatics analyses. Five promising candidate genes were identified (*LOC_Os05g40160*, *LOC_Os05g40180*, *LOC_Os12g36890*, *LOC_Os12g36900* and *LOC_Os12g36940*). The physiological traits of rice accessions that differed in terms of Fe toxicity tolerance suggest that ROS–scavenging antioxidant enzymes and Fe homeostasis are critical for alleviating the negative effects of Fe toxicity on rice. The study’s findings have enhanced our understanding of the genetic and physiological mechanisms underlying Fe toxicity tolerance in rice and may be exploited to further improve commercially cultivated rice varieties.

## Data Availability

The original contributions presented in the study are included in the article/Supplementary Material; further inquiries can be directed to the corresponding author.

## Supplementary Materials

The following supporting information can be downloaded at: https://www.mdpi.com/article/10.3390/ijms25136970/s1 [92,93,94,95].
