# Supplementary figures and images for: Candidate Genes and Favorable Haplotypes Associated with Iron Toxicity Tolerance in Rice
Source: Int J Mol Sci. 2024 Jun 26;25(13):6970. doi: 10.3390/ijms25136970 (PMC11241266; doi:10.3390/ijms25136970)

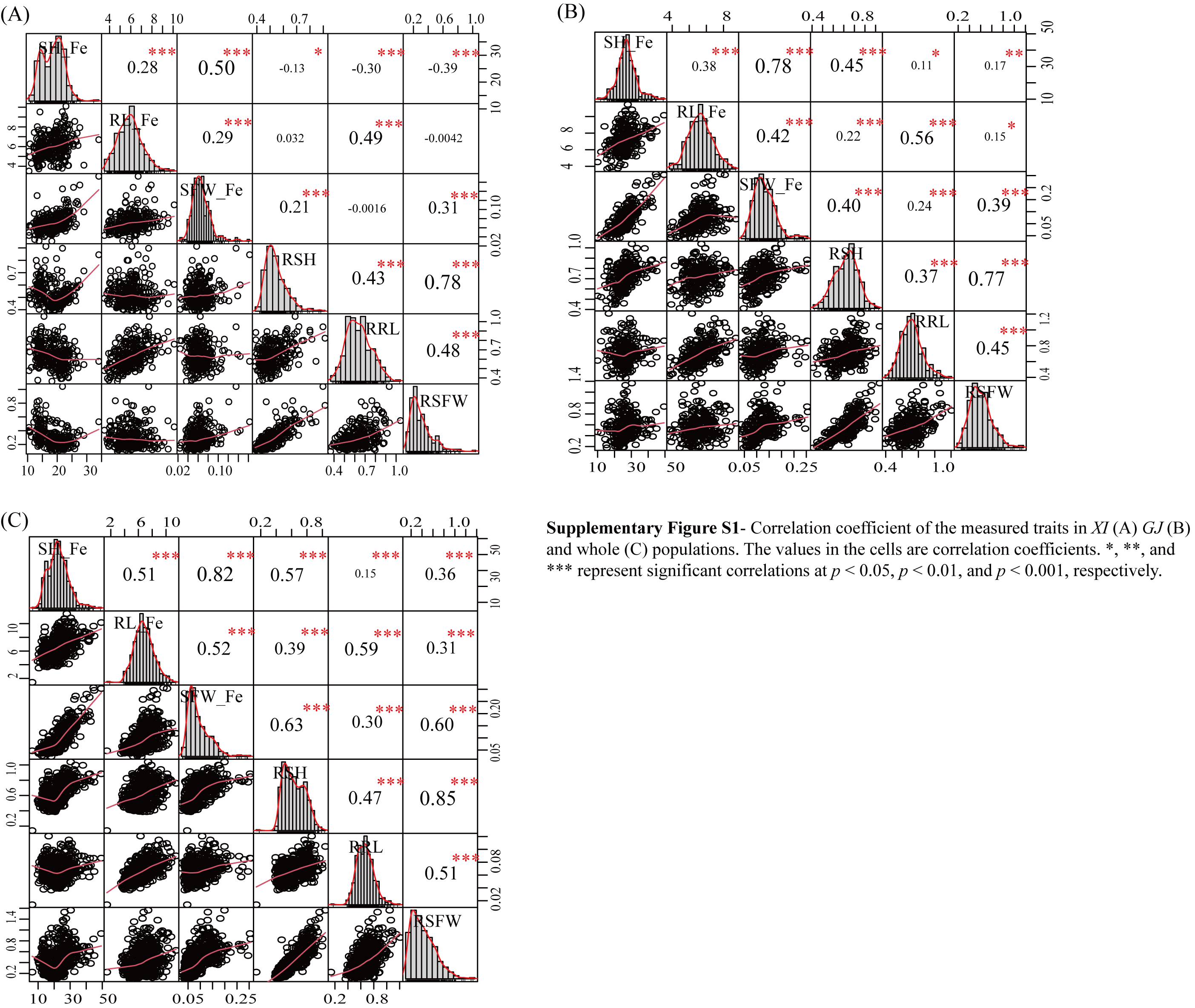

Supplement: Supplementary file 1 [file ijms-25-06970-s001.zip › Supplementary Figure S1.tif]

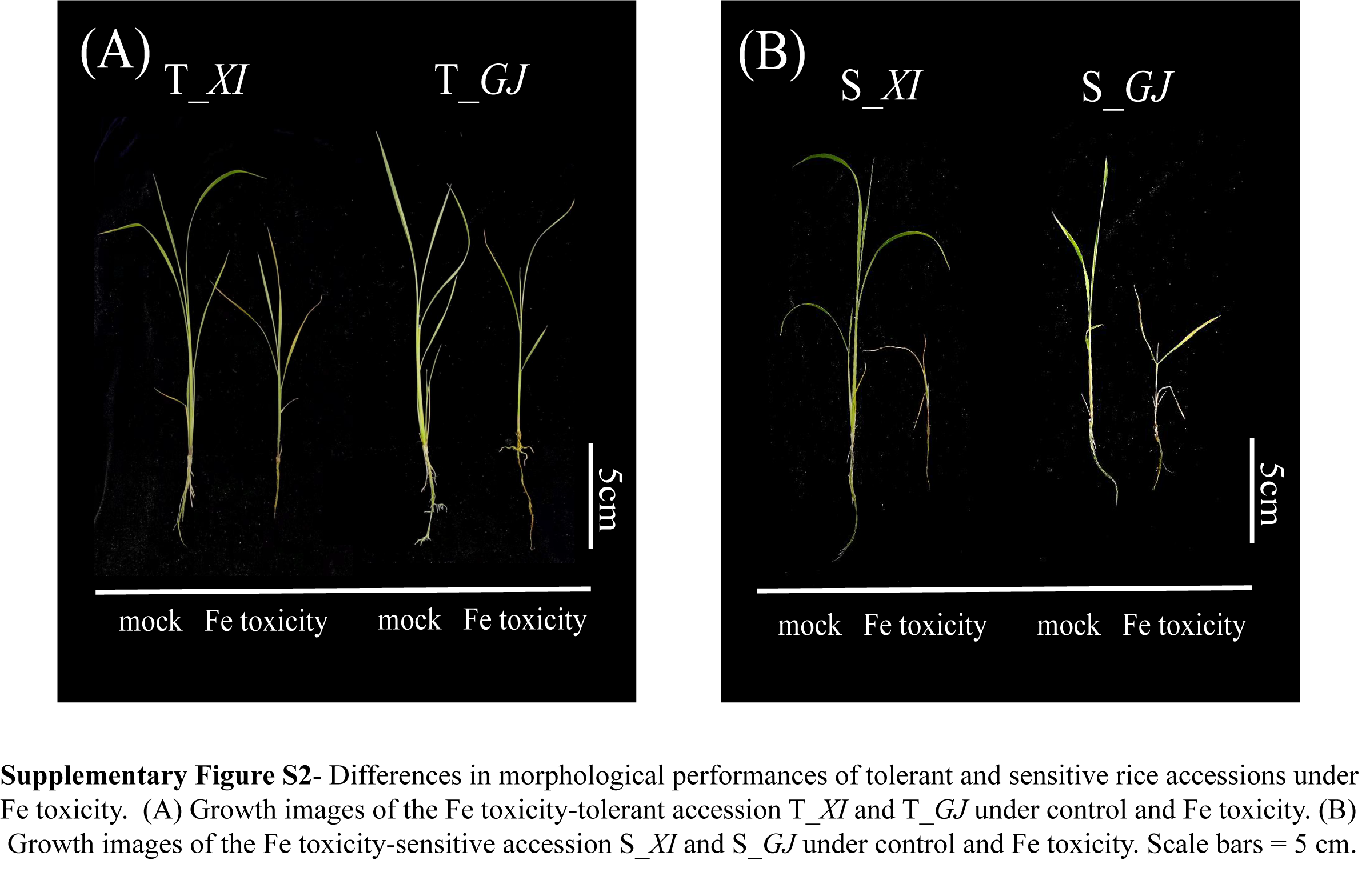

Supplement: Supplementary file 1 [file ijms-25-06970-s001.zip › Supplementary Figure S2.tif]

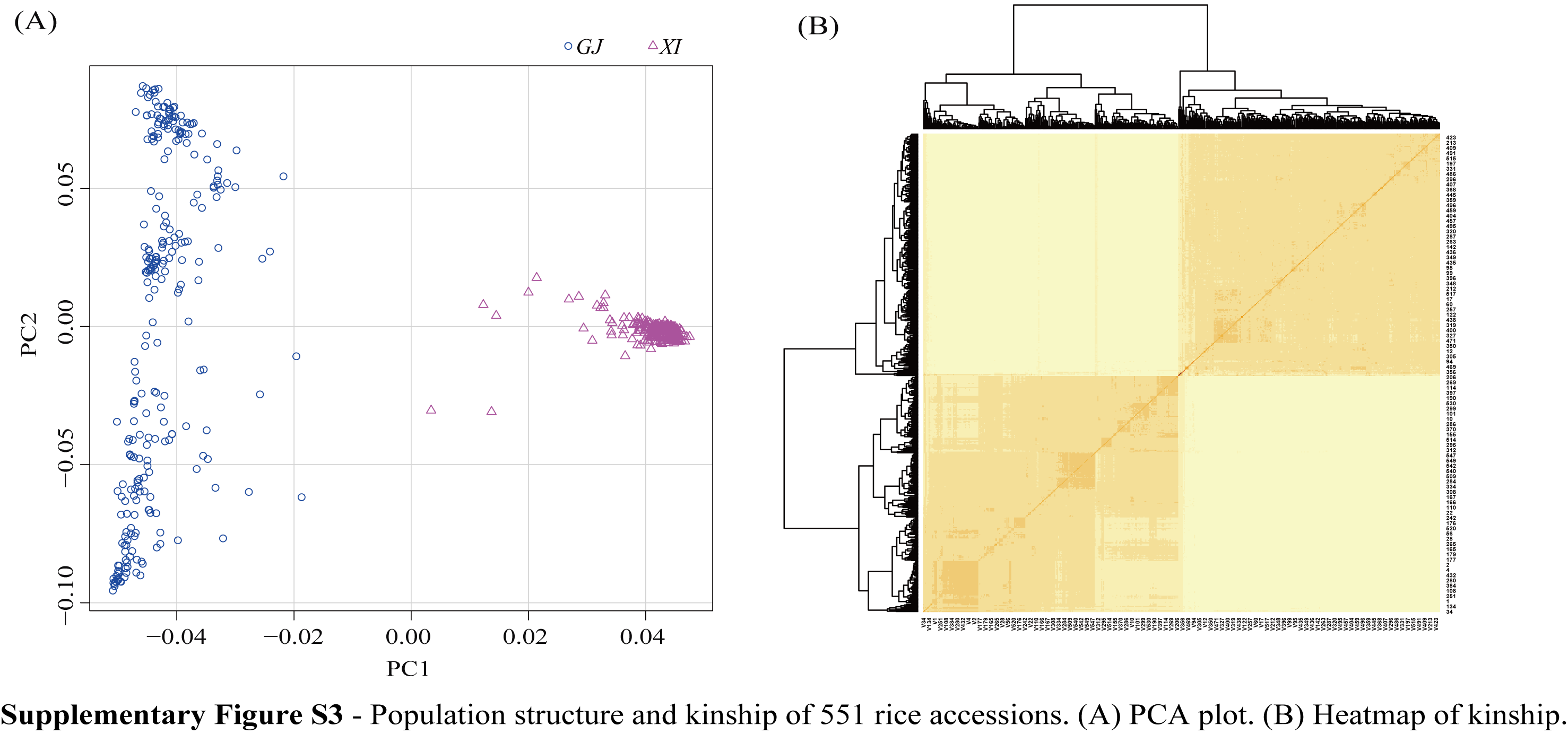

Supplement: Supplementary file 1 [file ijms-25-06970-s001.zip › Supplementary Figure S3.tif]

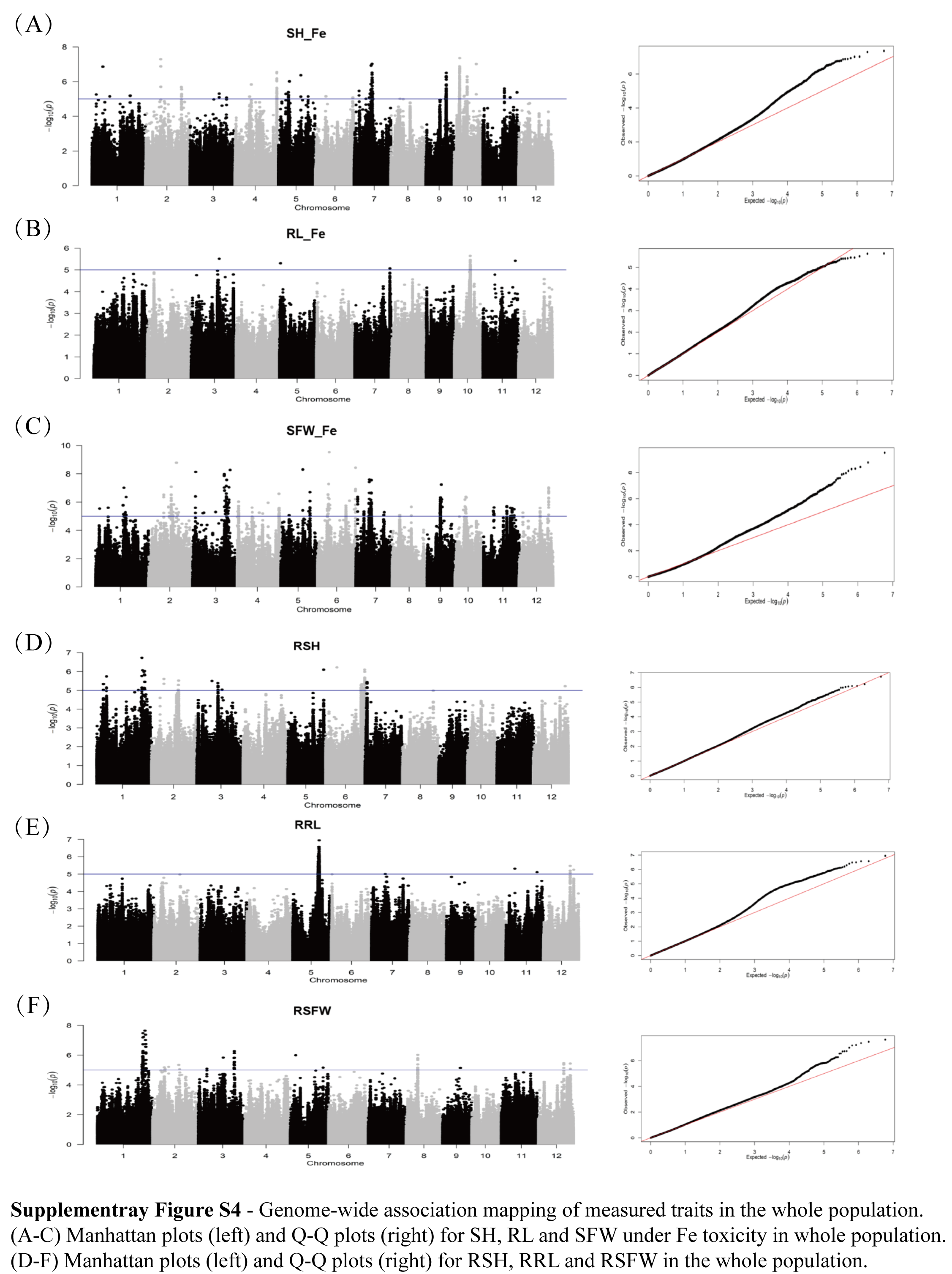

Supplement: Supplementary file 1 [file ijms-25-06970-s001.zip › Supplementary Figure S4.tif]

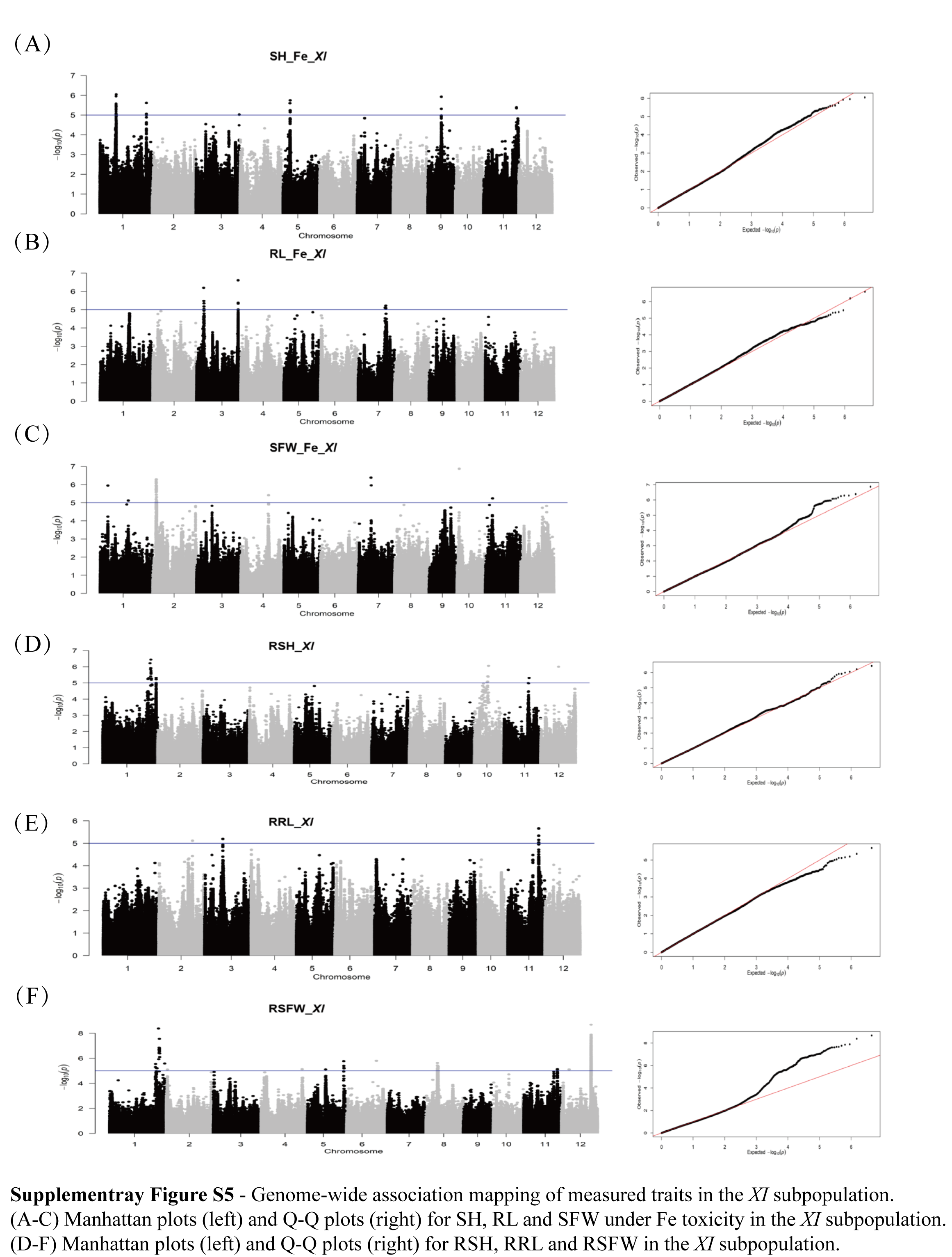

Supplement: Supplementary file 1 [file ijms-25-06970-s001.zip › Supplementary Figure S5.tif]

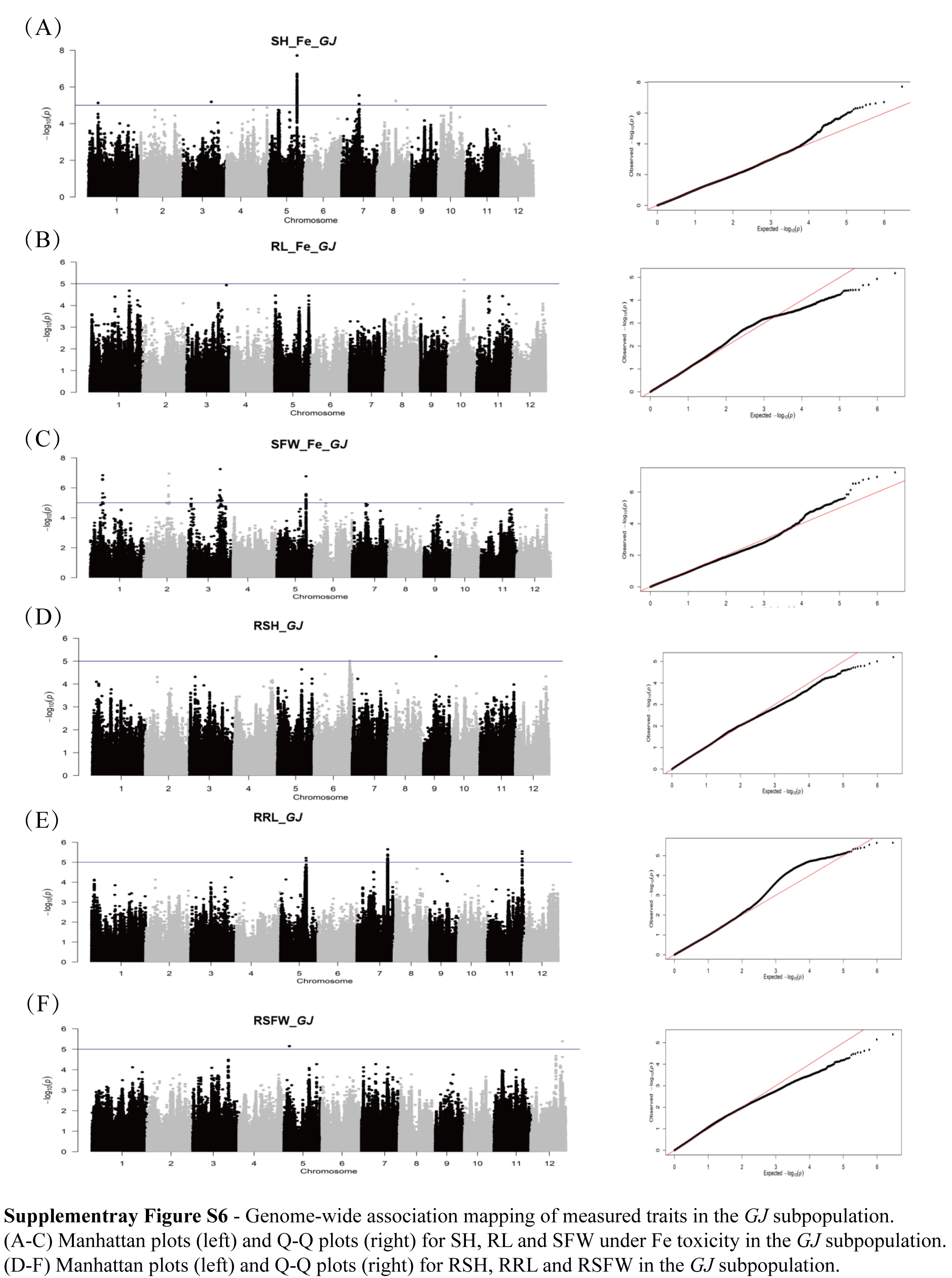

Supplement: Supplementary file 1 [file ijms-25-06970-s001.zip › Supplementary Figure S6.tif]
